# Incremental benefit of late gadolinium cardiac magnetic resonance imaging for risk stratification in patients with hypertrophic cardiomyopathy

**DOI:** 10.1038/s41598-017-06533-0

**Published:** 2017-07-24

**Authors:** Christina Doesch, Erol Tülümen, Ibrahim Akin, Boris Rudic, Juergen Kuschyk, Ibrahim El-Battrawy, Tobias Becher, Johannes Budjan, Arman Smakic, Stefan O. Schoenberg, Martin Borggrefe, Theano Papavassiliu

**Affiliations:** 1 1st Department of Medicine, University Medical Centre Mannheim, Mannheim, Germany; 2 Department of Radiology and Nuclear Medicine, University Medical Centre Mannheim, Mannheim, Germany; 3 DZHK (German Centre for Cardiovascular Research) partner site, Mannheim, Germany

## Abstract

Hypertrophic cardiomyopathy (HCM) has a low risk for sudden cardiac death (SCD). The ESC clinical risk prediction model estimates the risk of SCD using clinical and echocardiographical parameters without taking into account cardiac magnetic resonance (CMR) parameters. Therefore, we compared the CMR characteristics of 149 patients with low, intermediate and high ESC risk scores. In these patients left and right ventricular ejection fraction and volumes were comparable. Patients with a high ESC risk score revealed a significantly higher extent of late gadolinium enhancement (LGE) compared to patients with intermediate or a low risk scores. During follow-up of 4 years an extent of LGE ≥20% identified patients at a higher risk for major adverse cardiac arrhythmic events in the low and intermediate ESC risk group whereas an extent of LGE <20% was associated with a low risk of major adverse cardiac arrhythmic events despite a high ESC risk score ≥6%. Hence, we hypothesize that the extent of fibrosis might be an additional risk marker.

## Introduction

Hypertrophic cardiomyopathy (HCM) is a complex genetic heart disease^1–4^. Although the overall risk for sudden cardiac death (SCD) is relatively low, some patients with HCM die suddenly from fatal arrhythmic events^1, 5, 6^. Therefore, the identification of patients at risk for life-threatening arrhythmias is important. Based on risk stratification the implantation of implantable cardioverter defibrillator (ICD) in patients with HCM and a high risk has shown to reduce mortality^7–10^. However, in contrast to patients with ischemic heart disease treated with an ICD for primary or secondary prevention, patients with HCM are generally younger and predominantly present with a preserved left ventricular function. Furthermore, the prevalence of atrial fibrillation is higher in patients with HCM compared to other patient subgroups^11^. The younger age of the physically active HCM patients is accompanied by a higher rate of ICD lead failures^12^ and might result in further morbidity and mortality. The higher prevalence of atrial fibrillation represents an additional risk for inappropriate shocks in these patients^13^. Therefore, risk stratification in patients with HCM is crucial to avoid over- or undertreatment. The recently published ESC clinical risk prediction model^14^ uses clinical and echocardiographic determined parameters such as age, family history of SCD, maximal left ventricular wall thickness, left atrial diameter, left ventricular outflow tract gradient, previous unexplained syncope and the occurrence of non-sustained ventricular tachycardia to estimate the probability of sudden cardiac death (SCD) at 5 years. However, this risk prediction model is just based on a multicenter, retrospective longitudinal cohort study^15^ since prospective randomized studies are missing. Furthermore, the ESC risk prediction model does not take into account cardiac magnetic resonance (CMR) parameters. Therefore, the aim of our study was to compare patients with low, intermediate and high ESC risk scores of SCD according to CMR characteristics in order to identify additional imaging risk factors that might help to refine risk stratification.

## Methods

### Study population

A total of 149 patients from our HCM registry that underwent CMR examination between January 2008 and December 2015 at our department were included in the study. All patients gave informed consent and the study was approved by the local ethics commission (Medizinische Ethikkommmision II, Medizinische Fakultät Mannheim) and was performed in adherence to the Declaration of Helsinki. All patients with HCM were diagnosed based on conventional criteria; left ventricular hypertrophy ≥15 mm on two-dimensional echocardiography in the absence of another disease that could account for the hypertrophy^16^. Non-obstructive HCM (HNCM) was defined as a pressure gradient ≤30 mmHg at rest and after provocation. Patients with a pressure gradient >30 mmHg at rest or after provocation were classified as obstructive HCM (HOCM). Non-sustained ventricular tachycardia (nsVT) was defined as three or more ventricular extra systoles at a rate of ≥120 BPM, lasting less than﻿﻿ 30 seconds^14^.

### ESC risk score

ESC risk score was calculated according to the following formulas: Prognostic index = [0.15939858 × maximal wall thickness(mm)] − [0.00294271 × maximal wall thickness^2^ (mm^2^)] + [0.0259082 × left atrial diameter (mm)] + [0.00446131 × maximal(rest/Valsalva) left ventricular outflow tract gradient (mm Hg)] + [0.4583082 × family history SCD] + [0.82639195 × NSVT] + [0.71650361 × unexplained syncope] − [0.01799934 × age at clinical evaluation (years)] and Probability of SCD at 5 years = 1 − 0.998exp^(Prognostic index)^ ^14^. A risk of SCD at 5 years <4% was considered as low, ≥4–<6% as intermediate and ≥6% as high risk^14^.

### Image acquisition

All studies were performed using a 1.5 Tesla and 3.0 Tesla whole body imaging system (Magnetom Avanto and Skyra, Siemens Medical Systems, Healthcare Sector, Erlangen, Germany) using a six-element (Avanto) phased-array body coil and a 18-element body matrix coil (Skyra). Cine images were acquired using a retrospective-gated balanced segmented steady state free precession (trueFISP) sequence in three long-axis views (2-, 3-, and 4-chamber view) and in multiple short-axis views, covering the entire left ventricle (LV) from base to apex. Late gadolinium enhancement (LGE) images were obtained 10–15 min after intravenous administration of 0.2 mmol·kg^−1^ Gadoteric acid (Dotarem, Guerbet, Roissy CdG Cedex, France, Germany), using either an inversion recovery turbo Fast Low Angle Shot sequence or a phase-sensitive inversion recovery true fast imaging with steady state precission sequence at the same position as the long and short-axis cine acquisitions in end diastole^17^. The inversion time was adjusted per patient to optimally null signal from normal myocardium typically between 250 and 300 ms.

### CMR Image analysis

Left ventricular mass and volumes^18^ as well as right ventricular^19^ and left atrial volumes^20^ were determined using CMR as previously described. Right atrial volumes were evaluated on the 4-chamber view and/or a right ventricular 2-chamber view using the biplane area length method^21^. MAPSE measurements were assessed on 4-chamber view cine images. The distance between the basal septal mitral annulus (septal MAPSE) and a reference point outside the heart were measured in end-diastole and end-systole. Septal MAPSE was calculated by subtracting the end-systolic (ESL) from the end-diastolic length (EDL).

To determine TAPSE, the distance between the cutting edge of the tricuspid annulus with the RV free wall and a reference point outside the RV apex were measured in end-diastole (EDL) and end-systole (ESL). The point outside the RV apex was chosen in extension to the right ventricular apex and had to stay unchanged at end-diastole and end-systole. TAPSE was defined as the difference between EDL and ESL. LGE was assessed by a semi quantitative score as previously described^22^. One representative basal, midventricular and apical slice which revealed visually the greatest amount of LGE was chosen for analysis. Then, each segment was visually scored by an experienced observer for the total amount of LGE, irrespective of distribution throughout the width of the segment (segmental extent of LGE) using the following scoring scheme: 0 = 0%, 1 = 1–25%, 2 = 26–50%, 3 = 51–75% and 4 = 76–100%. By summing all the 17-segmental scores the total size of myocardial LGE was calculated for the entire left ventricle. The resulting summed score for the total left ventricle was thereafter expressed as a percentage of the maximum possible score.

### Follow-up and definition of study endpoints

Follow-up data for non-cardiac and cardiac death was analysed retrospectively by review of the corresponding medical records in our electronic Hospital Information System or by telephone interview with the patient or the treating family doctor in all 149 patients.

Among the 149 HCM patients included in the study, 120 (80.5%) patients were regularly examined at our outpatient clinic and a complete arrhythmic follow-up including regular holter ECG monitoring and complete ICD analysis could be provided. 29 (19.5%) were followed-up by referring cardiologist in private practice. For these 29 patients a complete documentation of arrhythmic events could not be provided and these patients were excluded from the arrhythmic follow-up. Another 3 (2.0%) patients were also excluded from analysis due to myomectomy shortly after MRI. Therefore, only 117 (78.5%) patients were included in the final analysis for major adverse cardiac events including sudden cardiac death (SCD), electrical storm, adequate ICD shock, adequate antitachycardia pacing (ATP), sustained ventricular tachycardia on holter electrocardiogram (ECG) or ECG.

The definition of cardiac event required the documentation of significant arrhythmia. ICD therapies were classified appropriate when they occurred in response to ventricular tachycardia or ventricular fibrillation. Electrical storm (ES) was defined as ≥3 separate ventricular tachycardia events ≤24 h. In case of out-of-hospital death not followed by autopsy, sudden unexpected death was classified as cardiac death.

### Statistical Analysis

All data are presented as a mean ± standard deviation. Continuous parameters were compared using a 2-tailed student’s t-test. The Mann-Whitney U test was applied for nonparametric data. Box plot analysis was used. All results were considered statistically significant when p < 0.05. Analyses were performed with Statistical Package for Social Sciences (SPSS for windows 14.0, Chicago, IL, USA).

Receiver operating characteristic (ROC) curves were used in the whole group of patients to find the optimal cut-off values for extent LGE% and probability of SCD according to the ESC risk score (with maximizing the sum of sensitivity and specificity) to predict major adverse cardiac arrhythmic events. Sensitivity was calculated in all patients with a low and intermediate ESC risk score (ESC risk score <6%) to evaluate how good an extent LGE ≥20% is at detecting a major adverse cardiac arrhythmic event. Sensitivity was calculated as number of true positives/(number of true positives + number of false negatives). In patients with a high ESC risk score (ESC risk score ≥6%) the negative predictive value of a LGE <20% was calculated to estimate the probability that despite a high ESC risk score the HCM patients will not suffer a major adverse cardiac arrhythmic event. The negative predictive value is defined as number of true negatives/(number of true negatives + number of false negatives). In patients with a low to intermediate ESC risk score (ESC risk score <6%) survival estimates and cumulative event rates were compared by the Kaplan–Meier method by using the time-to-first event for each endpoint. The time to event curves were estimated with the Kaplan–Meier method and compared with the log-rank test.

## Results

In our study cohort of 149 patients with HCM 121 (81%) belonged to the low, 18 (12%) to the intermediate and 10 (7%) to the high risk group. The probability of SCD at 5 years according to the ESC risk score^14^ was 1.8 ± 0.8% in the low risk, 4.6 ± 0.7% in the intermediate and 12.2 ± 8.1% in the high risk group. Table 1 presents the clinical characteristics as well as the distribution of the traditional risk factors according to the ESC risk groups. Compared to patients with a low and intermediate risk, those in the high risk group revealed the highest percentage of patients with hypertrophic obstructive cardiomyopathy (HOCM). Looking at the distribution of the traditional risk factors in the ESC risk groups we observed a significant lower percentage of syncope, SWT >30mm and family history of SCD in the low risk group compared to those patients with an intermediate or high ESC risk. Whereas the prevalence of non-sustained ventricular tachycardias showed a significant increase from the low to the intermediate and from the intermediate to the high risk group.

**Table 1:** Clinical characteristics and traditional risk factors according to ESC risk scores.

Parameter	Low ESC risk n = 121	Intermediate ESC risk n = 18	High ESC risk n = 10	p-value low vs intermediate ESC risk	p-value intermediate vs high ESC risk
Age	57 ± 14	48 ± 17	50 ± 12	0.01	0.71
Female gender	43/(36%)	2/(11%)	3/(30%)	0.04	0.21
HOCM	41/(31%)	6/(33%)	5/(50%)	0.96	0.39
Probability of SCD at 5 years	1.8 ± 0.8	4.6 ± 0.7	12.2 ± 8.1	<0.0001	0.0002
Syncope	18/(15%)	10/(56%)	8/(80%)	<0.0001	0.20
Family history of SCD	12/(10%)	4/(22%)	4/(40%)	0.13	0.32
SWT ≥30 mm	1/(1%)	3/(17%)	1/(10%)	0.0002	0.63
nsVT	8/(7%)	7/(39%)	9/(90%)	<0.0001	0.01

Data are presented as ± standard deviation.

Abbreviations: HOCM: hypertrophic obstractive cardiomyopathy, nsVT:non-sustained ventricular tachycardia, SCD:sudden cardiac death, SWT:septal wall thickness.

**Figure 1:**
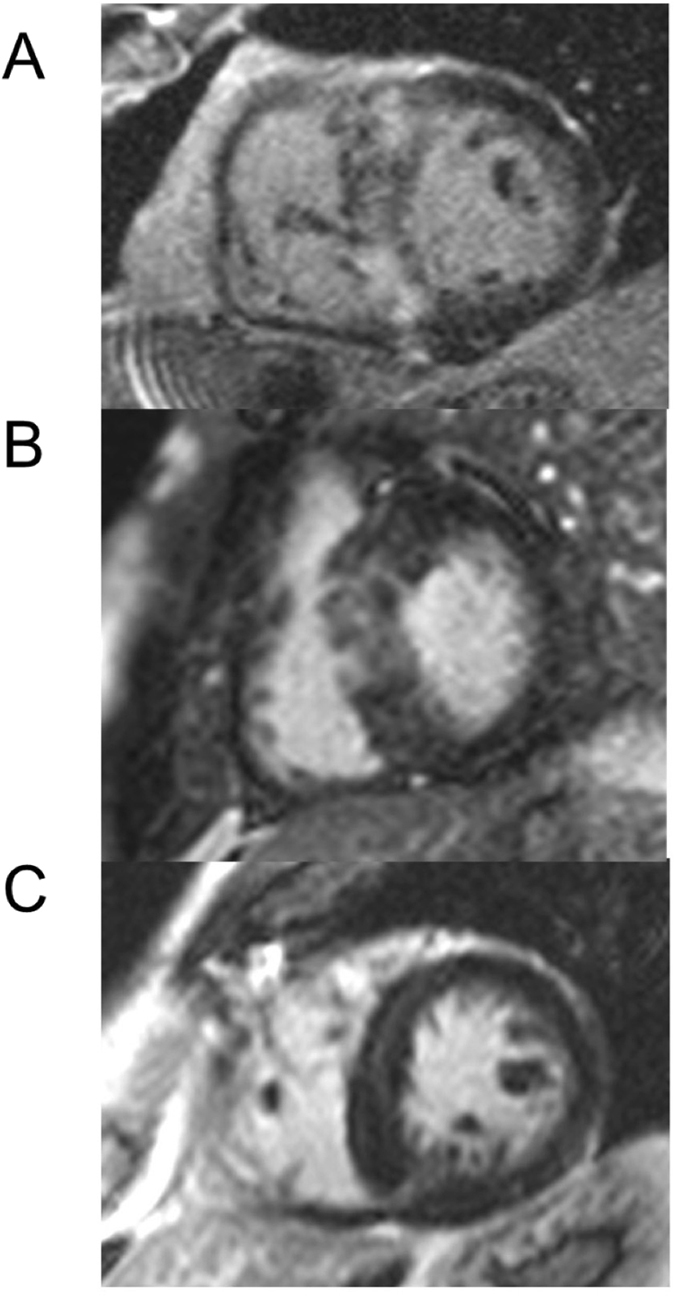
Image of a representative patients with a high ESC risk score and significantly more late gadolinium enhancement (panel A) than patients with intermediate ESC risk score (panel B) and low ESC risk score (panel C).

**Figure 2:**
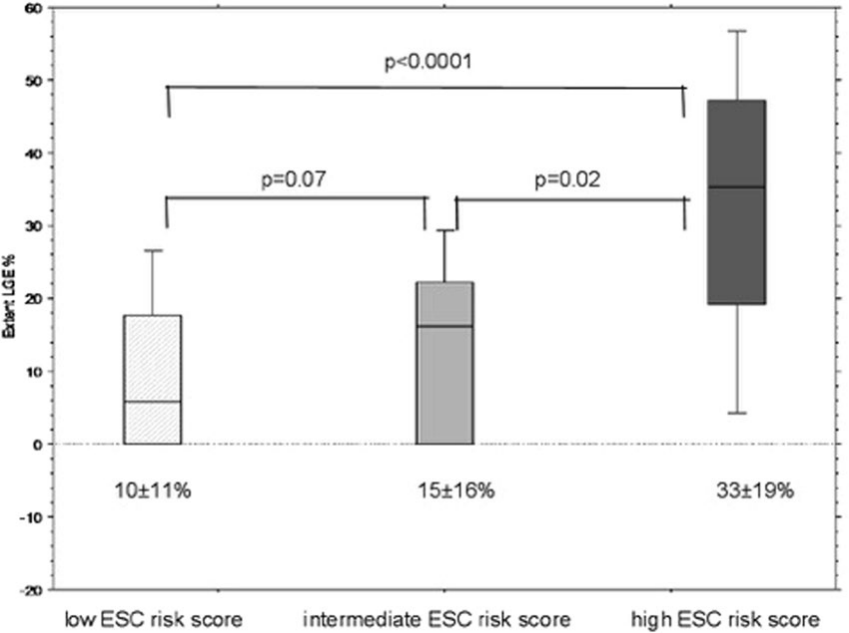
The box plots illustrate the extent LGE % in patients with a low ESC risk score (white crosshatched box plot), patients with intermediate ESC risk score (light gray box plot) and low ESC risk score (dark gray box plot). Extent LGE % is given as mean ± standard deviation below the box plots, the line in the box plots indicates the median value of the data. The p-values for the comparison of groups are also indicated. The figure also illustrates that patients with a high ESC risk score (defined as risk for SCD at 5 years ≥6%) reveal a significantly higher amount of LGE than those HCM patients with an intermediate (risk for SCD at 5 years ≥4–<6%) or low ESC risk score (risk for SCD at 5 years <4%). A risk of SCD at 5 years <4% was considered as low, ≥4–<6% as intermediate and as high risk. Abbreviations: HCM: hypertrophic cardiomyopathy, LGE: late gadolinium enhancement, SCD: sudden cardiac death.

Comparing CMR characteristics (Table 2), left and right ventricular ejection fraction as well as left and right ventricular volumes were comparable among the different risk groups. Patients with an intermediate and high ESC risk score had a significantly higher SWT (23 ± 6mm and 24 ± 5mm, respectively) compared to the low risk group (18 ± 4, Table 2). Whereas the left atrial end-diastolic volume index (LA-EDVI) was significantly higher in the high risk group (74 ± 45 ml/m²) compared to the intermediate (48 ± 17 ml/m²) and the low risk group (49 ± 30 ml/m²) (Table 2). However, patients with a high ESC risk score revealed a significantly higher extent of fibrosis defined as extent of late gadolinium enhancement (extent LGE %) 33 ± 19% (Fig. 1A, Fig. 2, dark grey bar) compared to patients with an intermediate risk score and an extent LGE % of 15 ± 16 (p = 0.02 Fig. 1B, Fig. 2 light grey bar) and patients with a low risk score and an extent LGE % of 10 ± 11% (p < 0.0001, Fig. 1C, Fig. 2 crosshatched bar).

**Table 2:** CMR characteristics according to ESC risk scores.

Parameter	Low ESC risk n = 121	Intermediate ESC risk n = 18	High ESC risk n = 10	p-value low vs intermediate ESC risk	p-value intermediate vs high ESC risk
LVEF (%)	61 ± 10	59 ± 12	58 ± 9	0.34	0.94
LVEDD (mm)	52 ± 7	54 ± 7	49 ± 5	0.28	0.05
LV-EDVI (ml/m²)	78 ± 22	80 ± 23	70 ± 13	0.83	0.21
SWT (mm)	18 ± 4	23 ± 6	24 ± 5	0.0001	0.57
LV-EDMI (g/m²)	90 ± 28	104 ± 36	104 ± 34	0.06	0.98
MAPSE septal (mm)	0.9 ± 0.13	1.0 ± 0.4	0.8 ± 0.3	0.42	0.13
LA-EDVI (ml/m²)	49 ± 30	48 ± 17	74 ± 45	0.87	0.04
Presence of LGE	75/(62%)	12/(67%)	9/(90%)	0.70	0.17
Extent LGE %	10 ± 11	15 ± 16	33 ± 19	0.07	0.02
RVEF (%)	61 ± 10	63 ± 12	61 ± 7	0.52	0.71
RVEDD (mm)	42 ± 6	41 ± 9	40 ± 3	0.70	0.67
RV-EDVI (ml/m²)	70 ± 19	70 ± 17	65 ± 12	0.95	0.40
RAD (mm)	43 ± 8	45 ± 10	45 ± 7	0.54	0.96
RA-EDVI (ml/m²)	51 ± 15	52 ± 17	53 ± 20	0.68	0.87

Data are presented as ± standard deviation. Volumes are indexed to body surface area.

Abbreviations: LVEF = left ventricular ejection fraction, LVEDD = left ventricular end diastolic dimension, LVEDMI = left ventricular end diastolic mass index, LV-EDVI = left ventricular end diastolic volume index, LA = left atrial, LGE = late gadolinium enhancement, LV = left ventricular, MAPSE = mitral annular plane systolic excursion, PWT = posterior wall thickness, RAD = right atrial diameter, RVEF = right ventricular ejection fraction, SWT = septal wall thickness, RA-EDVI = right atrial end diastolic volume index.

During the median follow-up period of 4 years in the whole study group of 149 HCM patients 4 (2.7%) patients died due to non-cardiac cause: lung cancer (n = 1), breast cancer (n = 1), malignant melanoma (n = 1) and a haemorrhagic shock after a peripheral vascular intervention (n = 1). A cardiac death occurred in 12 (8.1%) patients due endocarditis (n = 1), SCD (n = 9) and HTX (n = 2). In the final analysis for major adverse cardiac arrhythmic events, 117 patients with a complete arrhythmic documentation were included. During the median follow-up period the likelihood to suffer from a major adverse arrhythmic events was 14.5%. Among the 17 HCM patients who suffered a major adverse arrhythmic event, SCD occurred in 9 patients, 2 patient suffered ES, 1 experienced an adequate ICD shock. Sustained VT on holter ECG was found in another 4 patients and 1 patient presented with torsade de point tachycardia at the emergency room.

**Figure 3:**
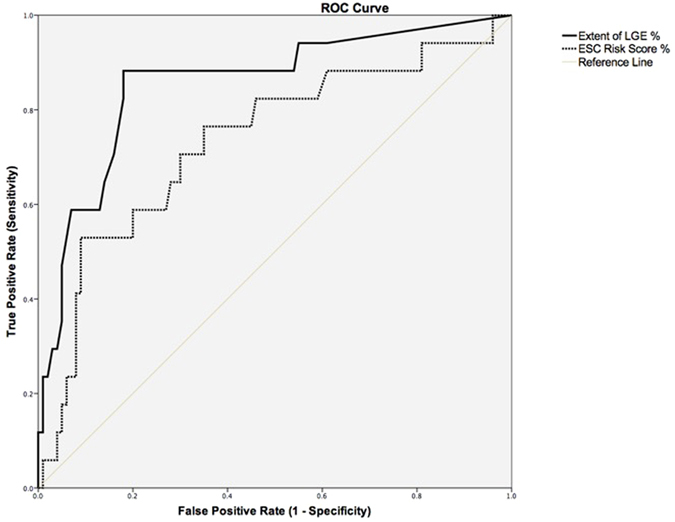
ROC Curves of extent LGE% and ESC risk score to predict major adverse cardiac arrhythmic events in patients with HCM. AUC for extent LGE% was 0.86 (95%CI 0.75–0.96; p < 0.0001) and for ESC risk score was 0.73 (95%CI 0.59–0.87; p = 0.002). ROC indicates receiver operating characteristic; AUC area under curves; HCM, hypertrophic cardiomyopathy; LGE, late gadolinium enhancement; CI, confidence interval.

**Figure 4:**
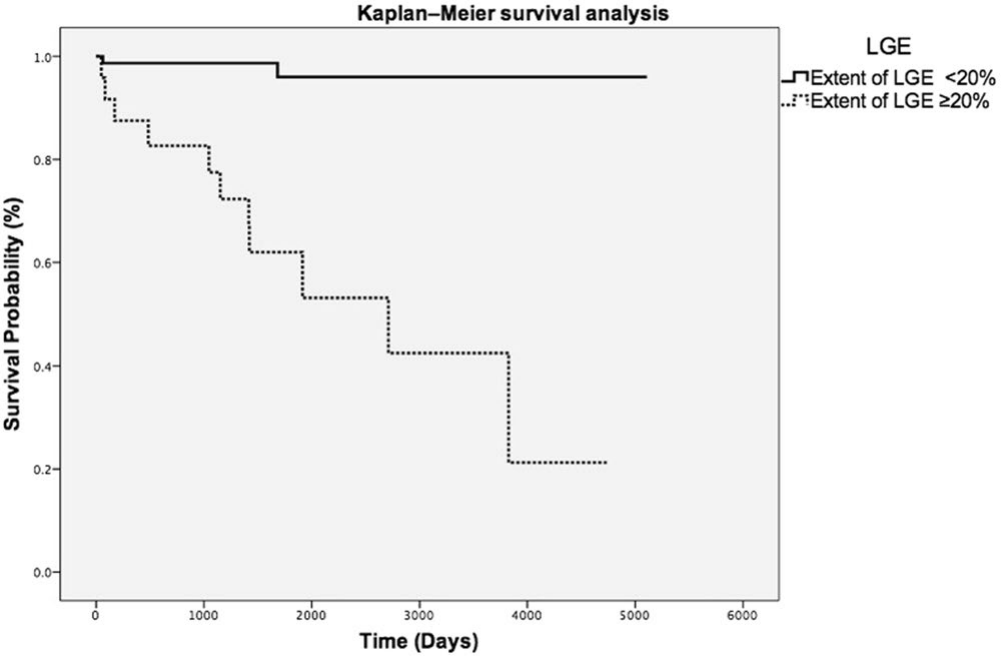
Kaplan-Meier event-free survival curves in patients with HCM according to extent of LGE%. According to this analysis, extent LGE ≥20% was an indicator of worse outcomes during follow-up compared with patients with extent LGE <20% (Log-rank, p < 0.0001). HCM indicates hypertrophic cardiomyopathy; LGE, late gadolinium enhancement.

ROC curves of extent LGE % showed an AUC of 0.86 (95%CI 0.75–0.96; p < 0.0001 (Fig. 3) and the optimal cut-off values for extent LGE% to predict major adverse cardiac arrhythmic events based on maximizing the sum of sensitivity and specificity was determined for an extent LGE of 20% (sensitivity: 88%; specificity: 82%). The ROC curve for the probability of SCD according to the ESC risk score revealed an AUC of 0.73 (95%CI 0.59–0.87; p = 0.002 (Fig. 3). The cut-off value for the probability of SCD according to the ESC risk score was 2.6% (sensitivity 71%, specificity 70%). Table 3 illustrates the distribution of patients with major adverse arrhythmic event and event-free patients according to ESC risk score classification in low, intermediate and high risk patients with HCM. Among the different risk groups, a further subdivision was created depending on the presence of an extent LGE ≥20% or <20%. This data shows that in the group of patients with a low or intermediate ESC risk score (<6%) the sensitivity of an extent LGE ≥20% to predict a major adverse arrhythmic event is 84.6%. The ability of extent LGE ≥20% for further risk stratification in the low and intermediate ESC risk group is further underlined by the significant higher event-rate in the group of HCM patients with an extent LGE ≥20% compared to HCM patients with an extent LGE % below the cut-off in Kaplan–Meier survival analysis (Fig. 4) (Log-rank, p < 0.0001). In the group of patients with an ESC risk <6% and an extent LGE ≥20% (n = 26), 11 major adverse cardiac arrhythmic events occurred during the follow-up period resulting in an annual event rate of 10.5% in these patients (Table 3). In patients with a high ESC risk score ≥6% none of the patients with an extent LGE <20% suffered from a major adverse arrhythmic event (Table 3). Therefore, an extent LGE <20% exhibits a negative predictive value of 100% in the ESC high risk group.

**Table 3:**
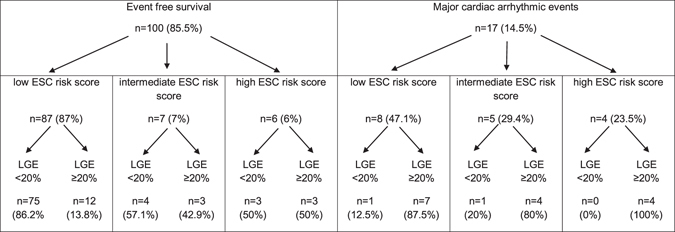
Distribution of patients according to ESC risk score and late gadolinium enhancement in CMR. *Abbreviations: ESC = European Society of Cardiology, LGE = late gadolinium enhancement, n = number*.

## Discussion

Our study showed that HCM patients with a high risk of SCD according to the ESC clinical risk prediction model revealed the highest extent of LGE % compared to patients with HCM and lower ESC risk scores. The extent LGE % showed a better predictive value to estimate major adverse cardiac arrhythmic events than ESC risk score. Furthermore, in patients with a low and intermediate ESC risk score <6% a high extent LGE % identified a subgroup of patients with an increased risk for major adverse cardiac arrhythmic events. On the other hand, in patients with high ESC risk score ≥6% an extent LGE <20% was associated with a low risk of major adverse cardiac arrhythmic events. Therefore, we concluded that these results support the hypothesis that the extent of LGE might play a role as an additional risk marker that could help to further improve risk stratification in these patients.

Most patients with HCM have a normal life expectancy^15, 23^. However, despite all efforts some patients with HCM still suffer from SCD^5^. Up to now, the risk for SCD was estimated clinically by evaluating parameters that reflect the severity of the underlying myocardial disease. In the last years, considerable improvements of risk stratification in these patients have been made. The new ESC risk prediction model^14^ is a validated risk prediction model for SCD that does not intend to render the physician’s judgement superfluous but helps to stratify patients to three risk groups with close interactions and smooth transitions treating the SCD risk as a continuum^15^.

Nevertheless, not all patients at risk are identified by conventional risk markers^24–26^. Therefore, intensive research activities have been undertaken to find new risk markers^27, 28^ aiming at identifying HCM patients who profit most from the implantation of a lifesaving ICD. Contrast enhanced CMR has emerged as an interesting additional imaging tool which is able to determine accurately the LV wall thickness due to high resolution imaging^29^ also in regions in which hypertrophy might be underestimated echocardiographically^30^. Moreover, CMR offers the possibility to detect areas of LGE. However, the precise pathophysiological basis for the occurrence of LGE in HCM is still ambiguous. Currently, LGE is mostly considered to represent areas of replacement fibrosis. This hypothesis is supported by two case reports^31, 32^ correlating areas of histological fibrosis to LGE in explanted hearts of patients with end-stage HCM. In the current literature, LGE is detected in 33–86% of patients with HCM^33–36^. The myocardial fibrosis is pathophysiologically assumed to be associated with re-entrant ventricular arrhythmia and myocardial dysfunction^37^. On holter monitoring, an association between LGE and NSVT could be shown^38, 39^. Besides, Fluechter *et al*.^40^ revealed that the extent of LGE % was higher in HCM patients with inducible ventricular tachyarrhythmias during programmed ventricular stimulation. Several small studies^35, 38, 39, 41, 42^ could show a correlation between the presence of LGE and adverse outcome in HCM, but there was only a trend towards an increased risk of SCD in the pooled data analysis^28^. The problem about the published data is the limited reliability due to selection and referral bias, differences in scanning protocols and LGE quantification^14^. Therefore, prospective multicenter longitudinal studies are needed to prove the role of the extent of LGE as an independent risk in patients with HCM. Due to the individually variable disease course in patients with HCM additional risk factors must therefore be established to decide which patients profit most form a more aggressive medical and device therapy.

The presence and extent of LGE seems to be a promising additional risk marker that has the potential to further improve the risk stratification in these patients beyond the known and well-established clinical risk markers.

## Conclusion

Patients with HCM who have a high ESC risk score reveal the highest extent of LGE compared to patients with HCM and an intermediate or low ESC risk score. During the follow-up an extent LGE ≥20% identified patients at a higher risk for major adverse cardiac arrhythmic events in the low and intermediate ESC risk group; whereas an extent LGE <20% was associated with a lower risk of major adverse cardiac arrhythmic events despite a high ESC risk score ≥6%. Therefore, the results of our study support the role of extent of LGE % as an additional risk marker that could help to further improve risk stratification in patients with HCM.

### Perspectives

In patients with HCM the identification of patients at risk for SCD is crucial. Drugs have not been proven to be effective to prevent SCD and ICD implantation is the only treatment strategy that has been shown to extend life. Therefore, risk stratification to identify the small proportion of patients who might benefit from prophylactic ICD implantation is a crucial part of the patient management in HCM. In contrast to patients with coronary artery disease, patients with HCM who undergo ICD implantation are young and might face serious ICD related complications during their lifetime. CMR using LGE has emerged as an important noninvasive imaging modality to visualize myocardial fibrosis that is supposed to be the underlying structural cause of the ventricular tachyarrhythmias. In our study, we could show that there is a significant increase in the amount of LGE with increasing clinical risk underlining the importance of visualizing and quantification of potential substrate. Furthermore, our study revealed a significantly higher risk for major cardiac arrhythmic events in patients with HCM and an extent LGE ≥20% belonging to the low and intermediate ESC risk score group. In these patients, a primary preventive ICD implantation would have been deferred based on the ESC risk prediction score but the additional determination of the extent LGE% identified a subset of patients at increased risk for major cardiac arrhythmic events. On the other hand, despite a high ESC risk score ≥6%, an extent LGE <20% seemed to identify a low risk patient group. None of these patients experienced a major cardiac arrhythmic event during follow-up of 4 years. Therefore, the absence of extent LGE might corroborate the decision to decide against ICD implantation in selected high risk patients.

Further standardized prospective multicenter and multivendor studies are needed to clearly depict the value of LGE CMR and establish the extent of LGE as a risk factor in future risk stratification models.
